# Transcriptional Heterogeneity of IgM^+^ Cells in Rainbow Trout (*Oncorhynchus mykiss*) Tissues

**DOI:** 10.1371/journal.pone.0082737

**Published:** 2013-12-06

**Authors:** Beatriz Abós, Rosario Castro, Jaime Pignatelli, Alfonso Luque, Lucia González, Carolina Tafalla

**Affiliations:** Centro de Investigación en Sanidad Animal (CISA-INIA), Valdeolmos, Madrid, Spain; Institut National de la Recherche Agronomique (INRA), France

## Abstract

Two major classes of B lymphocytes have been described to date in rainbow trout: IgM^+^ and IgT^+^ cells. IgM^+^ cells are mainly localized in the spleen, peripheral blood and kidney but are also found in other tissues. However, differences among IgM^+^ cell populations attending to its location are poorly defined in fish. Thus, the aim of this work was to characterize the expression of different immune molecules such as chemokine receptors, Toll-like receptors (TLRs) and transcription factors on sorted IgM^+^ lymphocytes from different rainbow trout tissues. IgM^+^ populations from blood, spleen, kidney, gills, intestine and liver were isolated by cell sorting and the constitutive levels of transcription of these genes evaluated by real-time PCR. To further characterize B cells, we identified an MS4A sequence. In humans, the MS4A family includes several genes with immune functions, such as the B cell marker CD20 or FcRβ. Subsequently, we have also evaluated the mRNA levels of this MS4A gene in the different IgM^+^ populations. The relevant differences in transcriptional patterns observed for each of these IgM^+^ populations analyzed, point to the presence of functionally different tissue-specific B cell populations in rainbow trout. The data shown provides a pattern of genes transcribed in IgM^+^ B cells not previously revealed in teleost fish. Furthermore, the constitutive expression of all the TLR genes analyzed in IgM^+^ cells suggests an important role for these cells in innate immunity.

## Introduction

In teleosts, B cells mature in the head kidney, the main hematopoietic organ, which is also thought to behave as a secondary organ [1]. On the other hand, the spleen is the main secondary lymphoid tissue due to the lack of lymph nodes in teleosts and appears to be an important site for B cell activation and plasmablast formation [2]. Additionally, B cells account for more than 30-40% of the cells in peripheral blood, and are also known to be present in the intestine [3], skin [4]and gills [5]. However whether these B cell populations constitute phenotypically and functionally different subsets has still not been elucidated in any fish species. 

Although not present in all species, to date, three different immunoglobulins (Igs) have been reported in teleosts, namely IgM [6], IgD [7] and IgT [8], designated as IgZ in zebrafish (*Danio rerio*) [9]. In rainbow trout (*Oncorhynchus mykiss*), two different subpopulations of B cells have been identified, IgM^+^/IgD^+^/IgT^-^ (IgM^+^ cells) and IgM^-^/IgD^-^/IgT^+^ (IgT^+^ cells) [8], whereas in catfish (*Ictalurus punctatus*) IgD^+^/IgM^-^ populations have also been described [10]. IgM^+^ and IgT^+^ cells are all constitutively present in diverse rainbow trout tissues, although the ratios between them differ. Thus, IgT^+^ cells constitute 16–28% of all trout B cells in the blood, spleen, head kidney and peritoneal cavity, while IgT^+^ B cells represent the main B cell population (54.3% of all B cells) in the gut [11]. Moreover, upon infection with an intestinal parasite, IgT responses were predominant in the gut, while IgM responses were confined to the serum [11]. Thus, it seems that IgT^+^ cells are specialized in mucosal responses, although recent studies have observed systemic IgT responses to viral infections [12] and mucosal mobilization of IgM^+^ cells in the gut upon oral vaccination [13]. In any case, as B cells are present in diverse tissues, it seems probable that IgM^+^ and IgT^+^ cells in these different locations display differences in their developmental and activation stages, accordingly to their different roles in antigen sensing, presentation and effector roles. 

Chemokines are chemoattractant cytokines responsible for leukocyte trafficking within the organism in homeostasis and disease. They not only mediate leukocyte mobilization, but also regulate the immune responses of the recruited cells, signaling through chemokine receptors, members of a family of seven transmembrane domain G-coupled receptors. In rainbow trout, the sequences of various chemokine receptors have been reported to date: CCR6 [14], CCR7 [15], CCR9 [16], CCR9B [14], CCR13 [14], CXCR1 [17] and CXCR4 [16]. However, there is no information concerning the chemokines that signal through these receptors and the patterns of transcription have only been assessed in complete tissues and not in individual cells, with the exception of CCR7 shown to be strongly transcribed in spleen T cells [15]. 

Toll-like receptors (TLRs) constitute a family of receptors that recognize conserved pathogen-associated molecular patterns (PAMPs), thus responsible for pathogen sensing and triggering of the innate immune response. In mammals, it has been reported that activation of TLRs provokes effects on B cells at two different levels. First, activation of TLRs through their ligands has been shown to induce important effects on innate responses such as cytokine production and non-specific proliferation of B cells. For example, CpG-rich bacterial DNA stimulates B cells to non-specifically secrete cytokines and chemokines [18], change chemokine receptors [19] or proliferate [20]. Secondly, and probably as a consequence of this first activation, TLR stimulation in B cells results in an increased specific antibody production [21], and this constitutes the basis for their potential as adjuvants [22]. In mammals, thirteen different TLRs that recognize different PAMPs have been identified [23], but in B cells, the expression of TLRs varies among species. Thus, human B cells moderately express TLR-1, -6, -7, -9, and -10 and low levels of TLR2, while in mouse B cells TLR-1, -2, -4, -6, -7, -9, and -10 are highly expressed in all B cell subsets [24]. Sheep B cells express TLR-1, -2, -4, -6, -7 and -10, but do not express TLR-3 or TLR-5 and show very low transcription levels of TLR-8 and -9 [25]. Furthermore, in humans, blood memory B cells have higher TLR expression levels and consequently higher PAMP sensibility, whereas naive B cells only express high levels of TLRs in tonsils [24]. Even though TLRs from fish and mammals have distinct features, they also have many structural similarities. However, and despite the identification of many different sequences in diverse teleosts, there is still very few data available concerning TLR signaling in these species. To date, eight TLRs have been identified in rainbow trout, namely TLR-1, -2, -3, -5, -7, -8, -9 and -22 [26]. 

Thus, to further investigate the heterogeneity of B cells in teleosts, in this study, we have characterized the expression of different immune genes on IgM^+^ lymphocytes from different rainbow trout tissues, including blood, spleen, kidney, gills, intestine and liver. We have focused on chemokine receptors, TLRs as well as the transcription factors Pax5 and Blimp1 (B lymphocyte-induced maturation protein-1). Finally, we have identified a sequence coding for a member of the MSA4 family, analyzing its transcription levels in different IgM^+^ subsets and throughout larval development. The MS4A family comprises 16 genes in humans, most of which remain uncharacterized [27]. Within this family, CD20 (MS4A1) is a membrane protein present in B cells for which expression varies through cellular differentiation, with minimal expression on early pre-B cells and no expression on plasma cells. Other characterized members from this family are MS4A2 (FcRb), a signaling subunit of the high affinity IgE receptor (FcƐRI) [28] and MS4A3 (Htm4), expressed on the membranes of lymphoid and myeloid cells, functioning as an adaptor protein in cell cycle regulation [29]. Murine MS4A4B is expressed in T cells, where it has a role in Th1 development and modulation of T cell function and signaling [30,31]. Although orthologs of human MS4A genes have only been identified in mammals, different homologs have been found in diverse jawed vertebrates including zebrafish [32]. These studies will contribute to further understand B cell activity in teleost fish, constituting a first step towards the characterization of different B cell subsets. 

## Materials and Methods

### Ethics statement

The experiments described comply with the Guidelines of the European Union Council (2010/63/EU) for the use of laboratory animals and were previously approved by the Ethics committee from the Instituto Nacional de Investigación y Tecnología Agraria y Alimentaria (INIA).

### Fish

Healthy specimens of female rainbow trout (*Oncorhynchus mykiss*) of approximately 50-70 g were obtained from Centro de Acuicultura El Molino (Madrid, Spain). Fish were maintained at the Centro de Investigaciones en Sanidad Animal (CISA-INIA) laboratory at 14°C with a re-circulating water system and 12:12 hours light:dark photoperiod. They were fed twice a day with a commercial diet (Skretting, Spain). Prior to any experimental procedure, fish were acclimatized to laboratory conditions for 2 weeks and during this period no clinical signs were ever observed. 

### Tissue collection

Rainbow trout were killed by MS-222 overdose and blood was extracted with a heparinized needle from the caudal vein and diluted 10 times with Leibovitz medium (L-15, Invitrogen) supplemented with 100 I.U./ ml penicillin, 100 µg/ ml streptomycin, 10 units/ ml heparin and 5% fetal calf serum (FCS). Spleen, kidney, gills, intestine and liver were collected and placed in the buffer mentioned above after transcardial perfusion using teleost Ringer solution pH 7.4 with 0.1% procaine in order to remove all the circulating blood from the tissues. Single cell suspensions from spleen, kidney, gills and liver were obtained using 100 µm nylon cell strainers (BD Biosciences). The intestine samples were opened lengthwise, washed in PBS and cut into small pieces. The cell extraction procedure was started with one round of 30 min agitation at 4°C in L-15 medium with P/S and 5% FCS, followed by an agitation in PBS with 1 mM EDTA and 1mM DTT for 30 min. Finally, the tissues were digested with 0.15 mg/ ml of collagenase (Sigma) in L-15 for 1.5 h at 20°C. All cell suspensions were placed onto 30/ 51% discontinuous Percoll (GE Healthcare) density gradients and centrifuged at 500 x *g* for 30 min at 4°C. The interface cells were collected and washed twice in L-15 containing 5% FCS.

### Cell sorting

Purified leukocytes were resuspended in PBS and incubated for 30 min on ice with a specific anti-trout IgM antibody coupled to phycoerythrin (1.14) [33]. Following two washing steps, cells were resuspended in PBS and IgM positive cells were sorted using a BD FACSAria III (BD Biosciences), using first their FSC/SSC profiles (to exclude the granulocyte gate) and then on the basis of the fluorescence emitted by the sample. IgM^+^ and IgM^-^ cells were then collected in different tubes for RNA isolation. 

### Real time PCR analysis of sorted cells

Total cellular RNA was isolated from IgM^+^ and IgM^-^ sorted populations from the different tissues using the Power Sybr Green Cells-to-Ct Kit (Invitrogen) following manufacturer´s instructions. RNAs were treated with DNAse during the process to remove genomic DNA that might interfere with the PCR reactions. Reverse transcription was also performed using the Power Sybr Green Cells-to-Ct Kit (Invitrogen) following manufacturer´s instructions. To evaluate the levels of transcription of the different genes, real-time PCR was performed with a LightCycler^®^ 480 System instrument (Roche) using SYBR Green PCR core Reagents (Applied Biosystems) and specific primers (shown in Table 1). The efficiency of the amplification was determined for each primer pair using serial 10 fold dilutions of pooled cDNA, and only primer pairs with efficiencies between 1.95 and 2 were used. Each sample was measured in duplicate under the following conditions: 10 min at 95°C, followed by 45 amplification cycles (15 s at 95°C and 1 min at 60°C) and a dissociation cycle (30 s at 95°C, 1 min 60°C and 30 s at 95°C). The expression of individual genes was normalized to relative expression of trout EF-1α and the expression levels were calculated using the 2^-ΔCt^ method, where ΔCt is determined by subtracting the EF-1α value from the target Ct. Negative controls with no template were included in all the experiments. A melting curve for each PCR was determined by reading fluorescence every degree between 60°C and 95°C to ensure only a single product had been amplified. 

**Table 1:** Primers used in real time analysis.

**Gene**	**Name**	**Sequence (5’-3’)**	**Reference**
CCR6	CCR6-F	TGCAGAGGAAACAGTTAACAATTCA	[14]
	CCR6-R	CCAGTAAACCCAGGATACAGATGAC	
CCR7	CCR7-F	TTCACTGATTACCCCACAGACAATA	[15]
	CCR7-R	AAGCAGATGAGGGAGTAAAAGGTG	
CCR9	CCR9-F	TCAATCCCTTCCTGTATGTGTTTGT	[14]
	CCR9-R	GTCCGTGTCTGACATAACTGAGGAG	
CCR9B	CCR9B-F	AATATTTCCAACGTCTGAAACAGGA	[14]
	CCR9B-R	CTCACCCAGGACTTATCACACATTC	
CCR13	CCR13-F	GTTCTGTACAACGTCTGGAAGGATT	[14]
	CCR13-R	ATGGCCAAAGGAAGTAGAAAGAAGA	
CXCR1	CXCR1-F	CCTGATATCCAGAAGCTCTTTGTGT	[17]
	CXCR1-R	TTGCATCCAGCTCTATGATAATGAA	
CXCR4	CXCR4-F	GTGCATGTGATCTACACCATC	[16]
	CXCR4-R	GAGCTGTGGCAAACACTATGT	
TLR-1	TLR-1-F	CAGACGCCCTGTTGATGTTC	[ [56]]*
	TLR-1-R	CCTTCACAAGTTCCACCACG	
TLR-2	TLR-2-F	GATCCAGAGCAACACTCTCAACAT	Accession number CCK73195*
	TLR-2-R	CTCCAGACCATGAAGTTGACAAAC	
TLR-3	TLR-3-F	AGCCCTTTGCTGCCTTACAGAG	[57]
	TLR-3-R	GTCTTCAGGTCATTTTTGGACACG	
TLR-5mem	TLR-5mem-F	TTGACTTATCTTCCAACGGATTCA	[ [58]]*
	TLR-5mem-R	CTTTGAAATTGCTGAAACCAAATG	
TLR-7	TLR-7-F	TACAGCTTGGTAACATGACTCTCC	[59]
	TLR-7-R	CAACTCTCTGAGACTTGTCGGTAA	
TLR-8a2	TLR-8a2-F	CATCTATGTTCTCATCCAGCAACC	[ [59]]*
	TLR-8a2-R	GGTCCCCCTAATAGACAACCTCTT	
TLR-9	TLR-9-F	TCTTCATAGAGCTGAAGAGGCCTCA	[ [60]]*
	TLR-9-R	GTTCCCACTGAGGAGAAGTGTTTT	
TLR-22	TLR-22-F	TGGACAATGACGCTCTTTTACC	[61]
	TLR-22-R	GAGCTGATGGTTGCAATGAGG	
Pax5	Pax5-F	ACGGAGATCGGATGTTCCTCTG	[54]
	Pax5-R	GATGCCGCGCTGTAGTAGTAC	
Blimp1	Blimp1-F	AGCTGTCCAACCTCAAGGTCC	[54]
	Blimp1-R	TTGCGGCACACCTGGGCATTC	
MS4-A	MS4-A-F	GACAACAAACTGAACAAATGTCTGG	This paper
	MS4-A-R	GTAAAAGATGATCCCAGCACTGTCT	
mIgM	mIgM-F	CCTTAACCAGCCGAAAGGG	[54]
	mIgM-R	CCAACGCCATACAGCAGAG	
EF-1α	EF-1α-F	GATCCAGAAGGAGGTCACCA	[14]
	EF-1α-R	TTACGTTCGACCTTCCATCC	

^*^ indicates that although the sequence had been previously reported and fully characterized, new primers for real time PCR were designed in this study.

### Immunohistochemistry

To visualize IgM^+^ in the different tissues, spleen, kidney, gills, intestine and liver obtained from naïve non-perfused fish were fixed in Bouin’s solution for 24 h, embedded in paraffin (Paraplast Plus; Sherwood Medical) and sectioned at 5 µm. After dewaxing and rehydration, some sections were stained with hematoxylin–eosin in order to determine the levels of infiltration, apparent damages or pathological changes. A second set of sections was subjected to an indirect immunocytochemical method for detection of trout IgM using a monoclonal antibody kindly donated by Dr. Kurt Buchmann from the University of Copenhagen and Dr. Karsten Skjoedt from the University of Southern Denmark (Denmark) [5,34]. Endogenous peroxidase was inhibited after rehydration by 10 min incubation in 3% H_2_O_2_ in PBS. After a heat induced epitope retrieval in Tris-EDTA buffer pH 9.0 (800 w for 5 min and 450 w for 5 min in a microwave oven), the sections were pre-incubated in two different blocking solutions consisting of 2% BSA (bovine serum albumin; Sigma-Aldrich) in TBT (Tris buffer with 0.02% tween 20) at room temperature for 30 min and 10% normal goat serum in TBT for 30 min. Sections were then incubated with a primary antibody solution (dilution 1:150) overnight at 4°C. Following this incubation, unbound primary antibody was washed off using TBT. The tissues were covered with anti-mouse EnVision^TM^ System HRP labeled secondary antibody (Dako) and left for a 30 min incubation period at room temperature. Subsequently, tissues were washed three times with TBT and then incubated in AEC substrate [0.05M acetic acid buffer (pH 5) with 0.015% H_2_O_2_ and 0.4 g/l 3-Amino-9-ethylcarbazole (Alfa Aesar)] for 15 min and afterwards washed for 4 min in tap water. The specificity of the reactions was determined by omitting the primary antibody. Mayer’s haematoxylin (Dako) was used as nuclear counter stain, and mounting was conducted with Aquamount (Merck). Slides were examined with an Axiolab (Zeiss) light microscope.

### Identification of an MS4A gene in rainbow trout

The human CD20 (MS4A1) protein sequence was used as a query against expressed sequence tag (EST) databases from rainbow trout (*Oncorhynchus mykiss*) in the National Center for Biotechnology Information database (http://blast.ncbi.nlm.nih.gov/Blast.cgi) using tBLASTn searches. 

A rainbow trout EST that encoded a MS4A-like sequence was identified [accession number CA359045.1]. The sequence lacked a stop codon, therefore 3’RACE was performed to obtain the complete sequence using a cDNA obtained from peripheral blood leukocytes (PBLs), the 3' RACE System for Rapid Amplification of cDNA Ends from Invitrogen and the primers MS4A-3´RACE (5´ ctgtgtctcgtcattcgcctgcagagctgt 3´) and MS4A-3´RACE nested: (5´ agtgttcatcattgggaatcaaataccggt 3´). An overlapping fragment which contained the final segment of the MS4A coding sequence and the 3´ UTR was amplified. Primers were then designed to amplify the full coding sequence, which was again sequenced. 

The complete MS4A nucleotide sequence was analyzed within the ExPASy Molecular Biology server (http://us.expasy.org) and deposited in the GenBank under Accession number KF387726. Similarity searches were performed using the basic local alignment tool program (http://blast.ncbi.nlm.nih.gov/Blast.cgi) and the program TMHMM Server v. 2.0 was used to predict the protein structure (http://www.cbs.dtu.dk/services/TMHMM/). Further phylogenetic analyses were performed using MegAlign Software (DNAstar Inc., Madison, USA) and the ClustalW algorithm. Phylogenetic trees were built using the neighbor-joining method and statistical parameters were determined by bootstrap analysis using 1000 replicates.

### cDNA preparation and real time PCR at early life stages

To investigate if MS4A is expressed at an early life stage, the ontogeny of MS4A expression was examined, in parallel to that of Blimp1, Pax5 and IgM. For this, eyed eggs at different degree days (DD) post-fertilization (~306 DD, ~354 DD, ~402 DD), immediate post hatch fry (hatch, ~450 DD), pre first feeding fry (PFF, ~562 DD), fry at the stage of full disappearance of the yolk sac (first feeding, FF, ~674 DD), and fry three weeks following first feeding (Fry, 786 DD) were sampled. The fish were always maintained at 16 °C in recirculated water.

Total RNA was extracted from the whole individuals sampled at different stages of the development using a combination of Trizol (Invitrogen) and RNAeasy Mini kit (Qiagen). In summary, 1 ml of Trizol was added to the eyed eggs and hatch stages and 2 ml were used for the stages of PFF, FF and fry. Samples were mechanically disrupted in Trizol using a disruption pestle. Then, 200 µl of chloroform per ml of Trizol were added and the suspension centrifuged at 12000 x *g* for 15 min. The clear upper phase was recovered, mixed with an equal volume of 100% ethanol and immediately transferred to RNAeasy Mini kit columns. The procedure was then continued following manufacturer’s instructions, performing on-column DNase treatment. Finally, RNA pellets were eluted from the columns in RNase-free water and stored at -80°C until used. 

Two µg of RNA were used to obtain cDNA in each sample using the Bioscript reverse transcriptase (Bioline Reagents Ltd) and oligo (dT)_12-18_ (0.5 µg/ ml) following manufacturer´s instructions. The resulting cDNA was diluted in a 1:5 proportion with water and stored at -20°C.

Real-time PCR was performed with a LightCycler^®^ 480 System (Roche) using FastStart SYBR Green Master mix (Roche) as previously described for sorted cells. Differences in the relative expression level of the genes among the different stages of development were determined using the Pfaffl method [35], comparing the mean expression of each group to the mean expression of the earliest stage (~306 DD). Efficiency of the amplification was determined for each primer pair using serial 10 fold dilutions of pooled cDNA, performed in the same plate as the experimental samples. The efficiency was calculated as E = 10 ^(-1/s)^, where s is the slope generated from the serial dilutions, when Log dilution is plotted against ΔCT (threshold cycle number).

## Results

### Distribution of IgM^+^ lymphocytes in rainbow trout tissues

**Figure 1:**
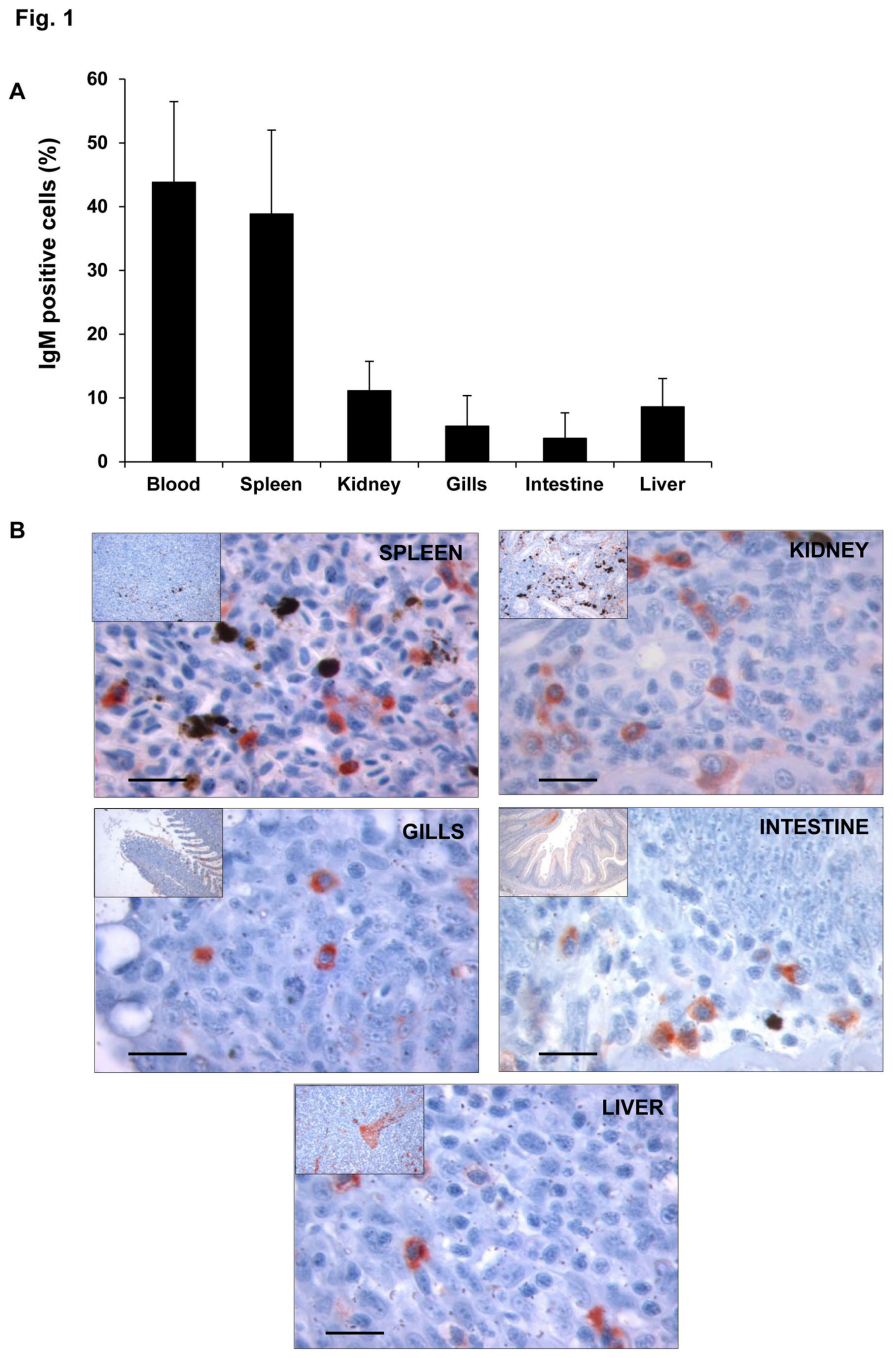
Location of IgM^+^ cells in diverse rainbow trout tissues. A. Mean percentage of IgM^+^ cells found in different trout tissues ± SD. Average from 6 to 11 individuals estimated by flow cytometry. B. Immunohistochemical detection of trout IgM^+^ cells in diverse trout tissues. The figures show a magnification image and an insert figure of the general location in which the detail was observed. Positive IgM detection is observed in red. Bar: 100 µm.

The main objective of this study was to analyze transcriptional differences for different IgM^+^ populations in rainbow trout. Therefore, our first step was to study the presence of IgM^+^ cells in the different trout tissues. To exclude a possible contamination of myeloid cells in the IgM^+^ fraction, we first sorted the cells on the basis of their FSC/SSC profiles and then on the basis of fluorescence within this lymphocyte-like gate (Figure S1). The frequency of IgM^+^ cells was very variable among the different trout tissues. As shown in Figure 1A, peripheral blood and spleen showed the highest percentage of IgM^+^ cells from total leukocytes (total cells obtained after Percoll density fractionation) with averages of 44% and 39%, respectively. The frequency of IgM^+^ cells in the kidney ranged between 5.7% and 19.8%; while levels obtained in the liver were between 2.7 and 13.7%. In contrast, the lowest percentages were found in the gills (1.6-12.5%) and intestine (0.3-4.9%). The differences in the frequency of IgM^+^ cells in the tissues sampled could not be due to the presence of peripheral blood lymphocytes as the trout used in the experiments were perfused prior to the tissue sampling to remove the circulating blood from the tissues. Therefore this variability seems to represent individual differences among the individuals tested and suggests a mobility of IgM^+^ cells from and to these tissues. 

To further confirm that these IgM^+^ cells corresponded to cells located within the tissue, the distribution of IgM^+^ cells in these tissues was also assessed through immunohistochemical staining (Figure 1B). IgM^+^ cells were detected all along the stroma of the different tissues analyzed in concordance to the results obtained through FACS analysis. Only in the kidney, the percentages of IgM^+^ cells observed through immunohistochemistry were higher than those detected through flow cytometry, possibly due to the presence of large numbers of plasma and pre-B cells with IgM in the cytoplasm but no membrane IgM. 

### Chemokine receptor transcription in sorted IgM^+^ populations from rainbow trout tissues

**Figure 2:**
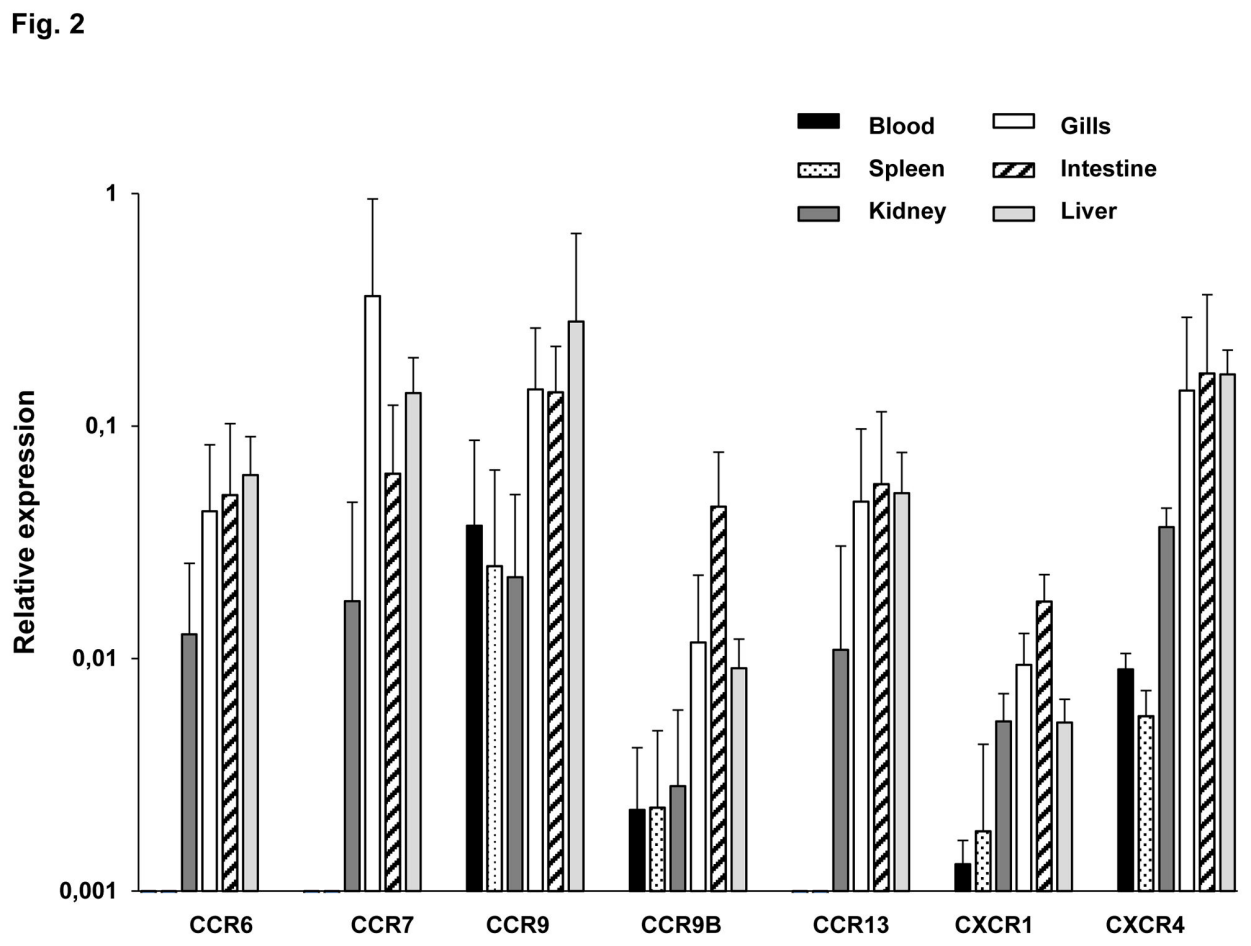
Levels of chemokine receptor transcription in sorted IgM^+^ cells. Constitutive levels of transcription of CCR6, CCR7, CCR9, CCR9B, CCR13, CXCR1 and CXCR4 in isolated IgM^+^ cells from different trout tissues. Levels of transcription were evaluated through real time PCR in duplicate. Data from 5 independent experiments are shown as the mean gene expression relative to the expression of an endogenous control (EF-1α) ± SD.

The expression of the chemokine receptors distinctively exposed on cells will characterize their migration pattern. Thus, to study whether different IgM^+^ cells from different tissues have a different migration potential, the levels of constitutive transcription of trout chemokine receptors in sorted IgM^+^ populations were determined through real time PCR analysis (Figure 2). All the tissues were obtained from fish after perfusion for depletion of circulating B cells. CCR6, CCR7 and CCR13 were expressed in kidney, gills, intestine and liver IgM^+^ cells, but not in IgM^+^ cells from blood or spleen. CCR9 and CCR9B were expressed in all IgM^+^ cells analysed, being CCR9B transcriptional levels lower than those found for CCR9. Concerning the CXC chemokine receptors analysed, both CXCR1 and CXCR4 were transcribed in all IgM^+^ populations, although the levels of transcription were much lower in blood and spleen. Generally, the transcription levels of all CC chemokine receptors were stronger in IgM^+^ cells isolated from gills and intestine than in IgM^+^ cells obtained from blood, spleen or kidney. The levels of transcription observed in liver were always similar to those found in gills and intestine with the exception of CCR9B and CXCR1. 

Within each tissue, we have also calculated the relations between the levels obtained in the positive fractions in comparison to those of the negative fractions, since these data could give us an indication of whether IgM^+^ cells accounted for most of the immune gene transcription in this tissue. As shown in Table 2, CCR7 transcription in sorted IgM^+^ cells from gills was almost 20 times higher than the transcription detected in sorted IgM^-^ leukocytes, indicating that this receptor is not only highly expressed in the gills, but it is mainly expressed in IgM^+^ cells. Moreover, IgM^+^ cells from gills also showed higher levels of transcription of CCR6, CCR9 and CCR13 than the IgM negative fraction, suggesting that IgM^+^ cells take account for the main transcription of the receptors in this organ. Highest expression of CCR9B was found in the IgM^+^ populations of kidney, intestine and liver (3 -13 times more than the negative fraction). Concerning the CXC receptors, elevated ratios of transcription between the positive and negative fractions were also observed in the intestine (for both CXCR1 and CXCR4) and in blood and spleen for CXCR4. 

**Table 2:** Ratio of chemokine receptor transcription in IgM^*+*^ cells in comparison to levels detected in the corresponding negative fractions (IgM^*+*^: IgM^-^).

	**CCR6**	**CCR7**	**CCR9**	**CCR9B**	**CCR13**	**CXCR1**	**CXCR4**
**Blood**	ND	ND	1,56	1,12	ND	1,27	2,73
**Spleen**	ND	ND	2,32	1,26	ND	0,74	5,33
**Kidney**	0,36	0,68	0,86	3,54	1,18	1,09	1,11
**Gills**	4,20	19,59	6,16	1,36	5,23	1,55	1,90
**Intestine**	1,60	0,60	0,62	3,20	1,12	5,92	2,61
**Liver**	0,96	1,60	1,43	13,10	0,93	1,54	1,94

Mean results from 5 experiments are shown.

Significant differences between positive and negative fractions are shown underlined (p < 0.05; Student´s *t* test). ND= transcription not detected in positive fractions.

These differences in chemokine receptor expression profiles and the differences in the ratios between positive and negative IgM cells suggest differences in the migration patterns of IgM^+^ cells to specific tissues. 

### TLRs transcription in sorted IgM^+^ populations from rainbow trout tissues

In order to assess tissue-specific expression of the described TLRs in isolated IgM^+^ cells from rainbow trout we also used real time PCR. All the TLR sequences used in this study had already been published (Table 1) with the exception of the TLR-2 found in the GenBank (Accession number CCK73195). Therefore, prior to the transcription analysis we verified that this sequence was in fact a true TLR-2 ortholog. The predicted trout TLR-2 sequence was used to search the GenBank protein database and the 50 top matches were TLR-2 sequences from diverse species. Additionally, an amino acid alignment of the trout TLR-2 amino acid sequence with fully characterized TLR-2 sequences already described in *Takifugu rubripes* [36], *Paralichthys olivaceus* [37] and *Epinephelus coioides* [38] revealed a high percentage of sequence identity (Figure S2). 

**Figure 3:**
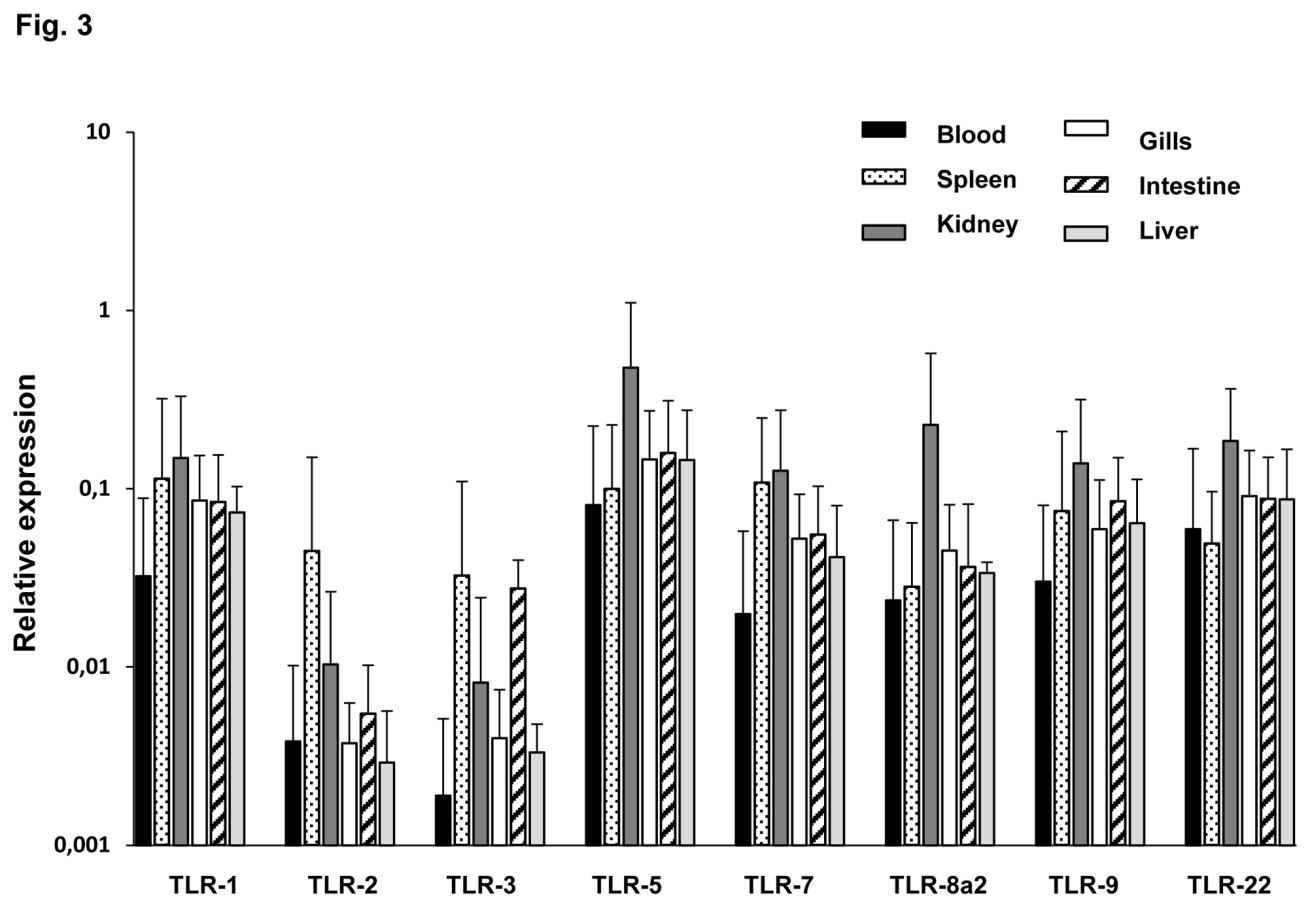
Levels of TLR transcription in sorted IgM^+^ cells. Constitutive levels of transcription of TLRs in IgM^+^ cells from different trout tissues. The amount of TLR-1, -2, -3, -5, -7, -8a2, -9 and -22 mRNA was evaluated through real time PCR in duplicates. Data from 5 independent experiments are shown as the mean gene expression relative to the expression of an endogenous control (EF-1α) ±SD.

As shown in Figure 3, all the TLRs analysed were constitutively transcribed by IgM^+^ cells from all tissues. However, TLR-2 and TLR-3 transcription levels were lower than levels observed for the rest of TLRs in all the tissues sampled except in IgM^+^ cells from spleen. On the other hand, IgM^+^ cells from kidney expressed higher levels of TLR-5, TLR-8a2, TLR-9 and TLR-22 than IgM cells from other tissues. Moreover, TLR-1 and TLR-7 transcription was slightly higher in IgM^+^ cells from spleen and kidney in comparison with the other tissues.

Table 3 shows the ratios of TLR expression between IgM^+^ and the corresponding negative fraction in the tissues analyzed. In blood, the transcription of TLR-2 in IgM^+^ cells was 7.5 times higher than the levels observed in the negative fraction, suggesting that, in blood, this receptor is mainly expressed in IgM^+^ cells. Regarding spleen and kidney, the levels of TLR-2 and TLR-3 in the positive fraction compared to the levels observed in the negative fraction, point to an almost exclusive transcription of TLR-2 and TLR-3 in the IgM^+^ cells in both organs. In general, gills, intestine and liver expressed higher TLRs transcription levels in sorted IgM^+^ cells in comparison with the negative fraction of leukocytes, with the exception of TLR-2 transcription levels in the gills. These results point to B cells as key players in the activation of immune responses in pathogen rich-areas like gills and intestine through TLRs and suggest an important role of the liver in pathogen-sensing. 

**Table 3:** Ratio of TLR transcription in IgM^*+*^ cells in comparison to levels detected in the corresponding negative fractions (IgM^*+*^: IgM^-^).

	**TLR-1**	**TLR-2**	**TLR-3**	**TLR-5**	**TLR-7**	**TLR-8**	**TLR-9**	**TLR-22**
**Blood**	0,19	7,50	0,49	0,31	0,26	0,22	0,39	0,40
**Spleen**	0,61	65,47	21,65	0,30	0,96	0,31	0,58	0,22
**Kidney**	0,73	6,00	18,16	0,62	0,51	1,13	0,59	0,49
**Gills**	1,98	0,47	2,66	4,50	4,47	5,05	5,91	4,47
**Intestine**	8,32	40,44	29,20	4,81	6,27	3,61	10,11	4,87
**Liver**	1,42	6,36	2,44	2,25	1,24	1,29	2,04	1,83

Significant differences between positive and negative fractions are shown underlined (p < 0.05; Student´s *t* test). Mean results from 5 experiments are shown.

### Transcription of Pax5 and Blimp1 in sorted IgM^+^ populations from rainbow trout tissues

**Figure 4:**
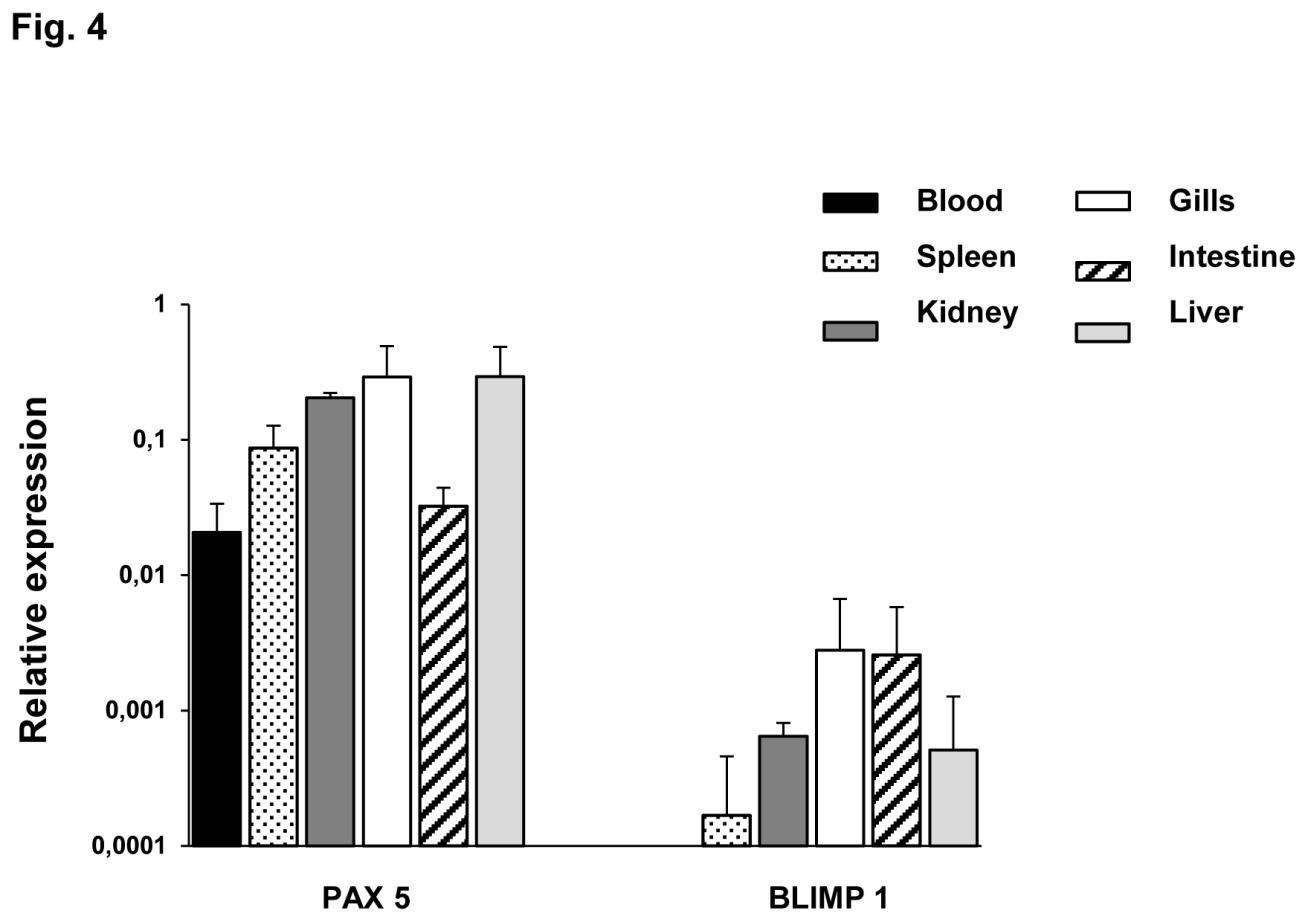
Levels of Pax5 and Blimp1 transcription in sorted IgM^+^ cells. Constitutive levels of transcription of Pax5 and Blimp1 in IgM^+^ cells from different trout tissues. Levels of transcription were evaluated through real time PCR in duplicates. Data from 3 independent experiments are shown as the mean gene expression relative to the expression of an endogenous control (EF-1α) ± SD.

To contribute to the characterization of the transcriptional heterogeneity among different IgM^+^ populations, we also studied the levels of transcription of Pax5 and Blimp1. Pax5 is a B cell-specific transcription factor down-regulated through the maturation of B cells due to the induction of the transcriptional repressor Blimp1 [39]. As expected, the levels of transcription of Pax5 were much more elevated than those observed for Blimp1 (Figure 4), suggesting that most IgM^+^ cells are in a non-activated state in naïve fish. Pax5 transcription was lower in blood and intestine IgM^+^ cells than in IgM^+^ cells from other tissues. Regarding Blimp1, there was no Blimp1 transcription in blood, whereas high levels were observed in gills and intestine. This result may indicate the presence of more mature IgM^+^ populations in these mucosal tissues. 

Table 4 shows the ratios of Pax5 and Blimp1 expression between sorted IgM^+^ and the corresponding negative fraction in the tissues analyzed. As expected, the ratios of Pax5 observed among different tissues correlate quite well with the ratios found for the transcription of the membrane form of IgM. These data suggest that IgM^+^ cells account for most of the Pax5 and Blimp1 transcription in the different tissues, since only a low ratio in comparison to the corresponding negative fraction was obtained for Pax5 in blood. The reason for this is currently unknown. 

**Table 4:** Ratio of MS4A, Pax5 Blimp1 and membrane IgM (mIgM) transcription in IgM^*+*^ cells in comparison to levels detected in the corresponding negative fractions (IgM^*+*^: IgM^-^).

	**MS4A**	**Pax5**	**Blimp1**	**mIgM**
**Blood**	0,029	0,84	ND	5,75
**Spleen**	0,67	2,87	1,67	15,33
**Kidney**	2,16	9,81	62,92	21,88
**Gills**	0,70	153,51	7,00	358,29
**Intestine**	6,64	53,88	12,86	22,11
**Liver**	0,16	16,95	5,11	45,79

Significant differences between positive and negative fractions are shown underlined (p < 0.05; Student´s *t* test). Mean results from 5 experiments are shown. ND= transcription not detected in positive fractions.

### Identification of an MS4A sequence in rainbow trout

**Figure 5:**
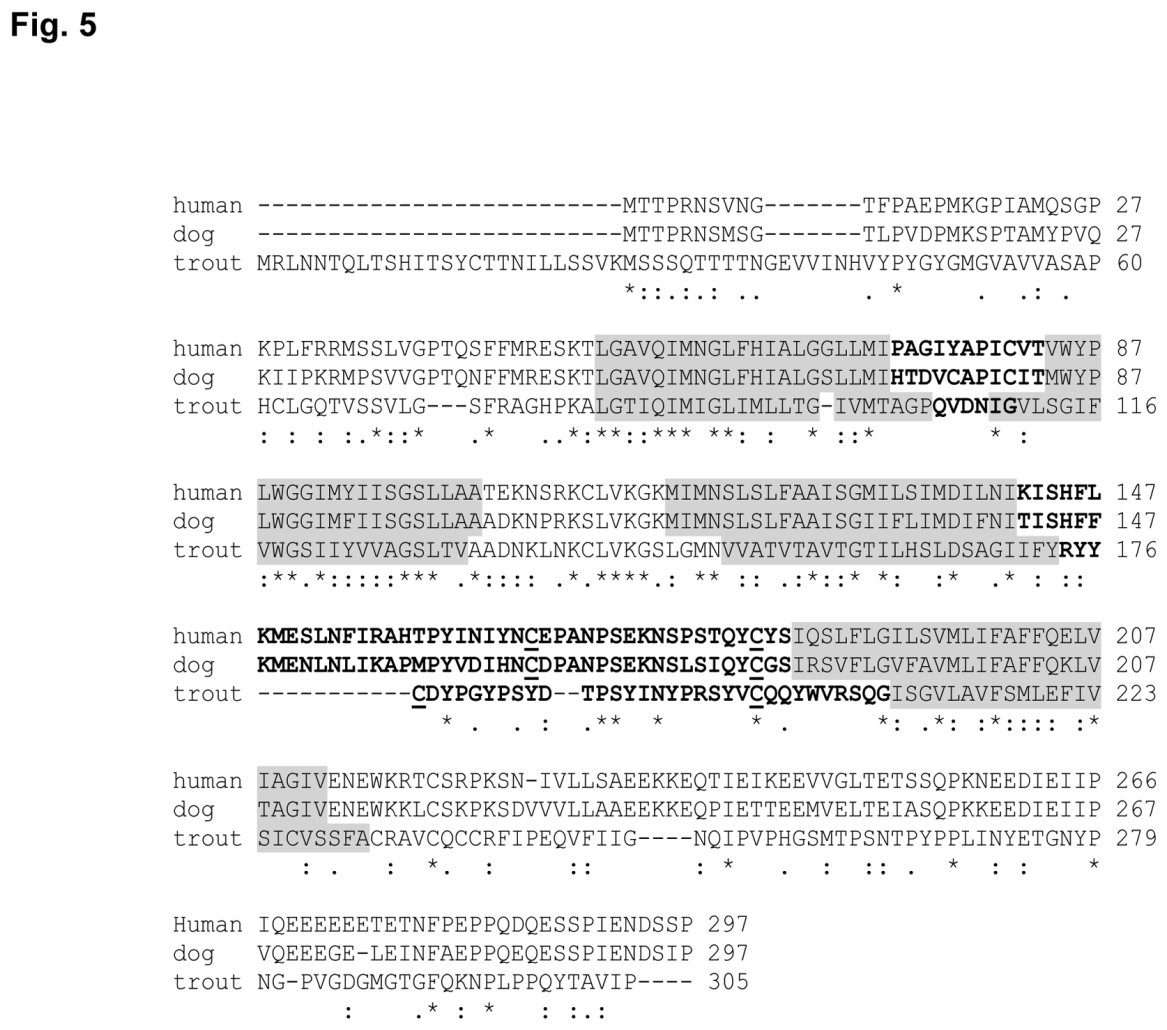
Identification of a rainbow trout MS4A family member. Alignment of predicted rainbow trout MS4A amino acid sequence with CD20 genes in human (NP_690605) or dog (BAE47068.1). Shaded grey residues show predicted transmembrane regions, while bold regions denote predicted extracellular domains. Cystein residues predicted to form a disulphide bond in the second extracellular loop are underlined. Identical residues from the aligned sequences are indicated by “*” below the alignment. Similar residues are marked by “.” or “**:**” below the alignments.

**Figure 6:**
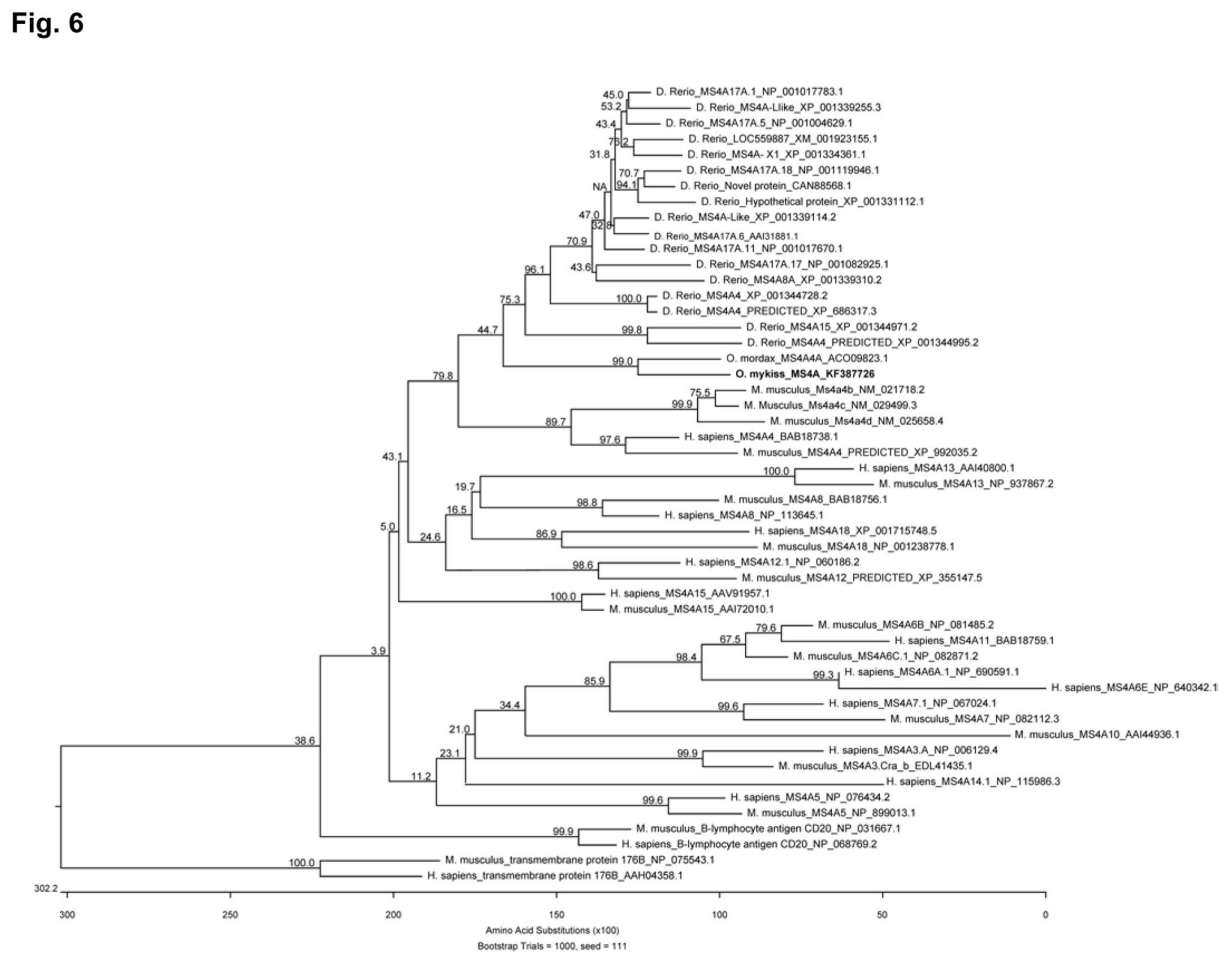
An unrooted phylogenetic tree was constructed using the trout MS4A amino acid sequence (bold) and available published MS4A amino acid sequences from mammals and fish. The tree was constructed using an amino acid multiple alignment using the ClustalW algorithm and the neighbor joining method. Bootstrap values (1000 replicates) are shown above the branches.

To further characterize B cells, we used the human CD20 to search the databases for a potential homolog in rainbow trout. Although we identified a MS4A family member that closely resembles CD20, it will not be possible to establish it as an equivalent gene until further additional functional assays are performed and this is why we decided to designate it as MS4A. The predicted MS4A rainbow trout protein sequence was used to search the GenBank Protein database using the DELTA-BLAST Algorithm. This search identified that the sequence had a CD20 family member signature and was most similar to mammalian CD20 than to other mammalian family members. Selected top matches are shown in Figure S3. The putative rainbow trout MS4A cDNA isolated from PBLs contained an open reading frame (ORF) of 1319 bp encoding a protein of 305 aa (Figure 5). The predicted amino acid sequence of rainbow trout MS4A revealed four major hydrophobic regions of approximately 22 amino acids, three intracellular and two extracellular domains as predicted for CD20 in mammals [40,41]. The first cytoplasmic region of the protein is longer in rainbow trout, whereas the first loop between helix one and two is extremely small, even smaller than in the human or canine sequence, thus seems unlikely that it can protrude extensively out of the membrane. On the other hand, the second extracellular loop between helix three and four is approximately 34 amino acids in length containing a possible disulphide bond between two cysteine residues, as was also predicted for mammalian CD20 [42]. In mammals, the MS4A family includes at least 24 distinct human and mouse genes with a striking conservation of the first three transmembrane domains [27,43], whereas 21 different MS4A genes have been recently identified in zebrafish [32]. Therefore, to further confirm its identity, we constructed a phylogenetic tree based on a multiple alignment of different MS4A gene sequences from fish and mammals (Figure 6; Figure S4). The trout MS4A was highly related to other fish sequences, specially to an MS4A gene sequence identified in rainbow smelt (*Osmerus mordax*), but grouped separately from MS4A genes in other vertebrate classes, as previously observed for the MS4A genes described in zebrafish [32]. These results suggest that in fish these genes diversified relatively recently from a common ancestor gene.

### Distribution of MS4A expression in rainbow trout tissues and isolated IgM^+^ cells

**Figure 7:**
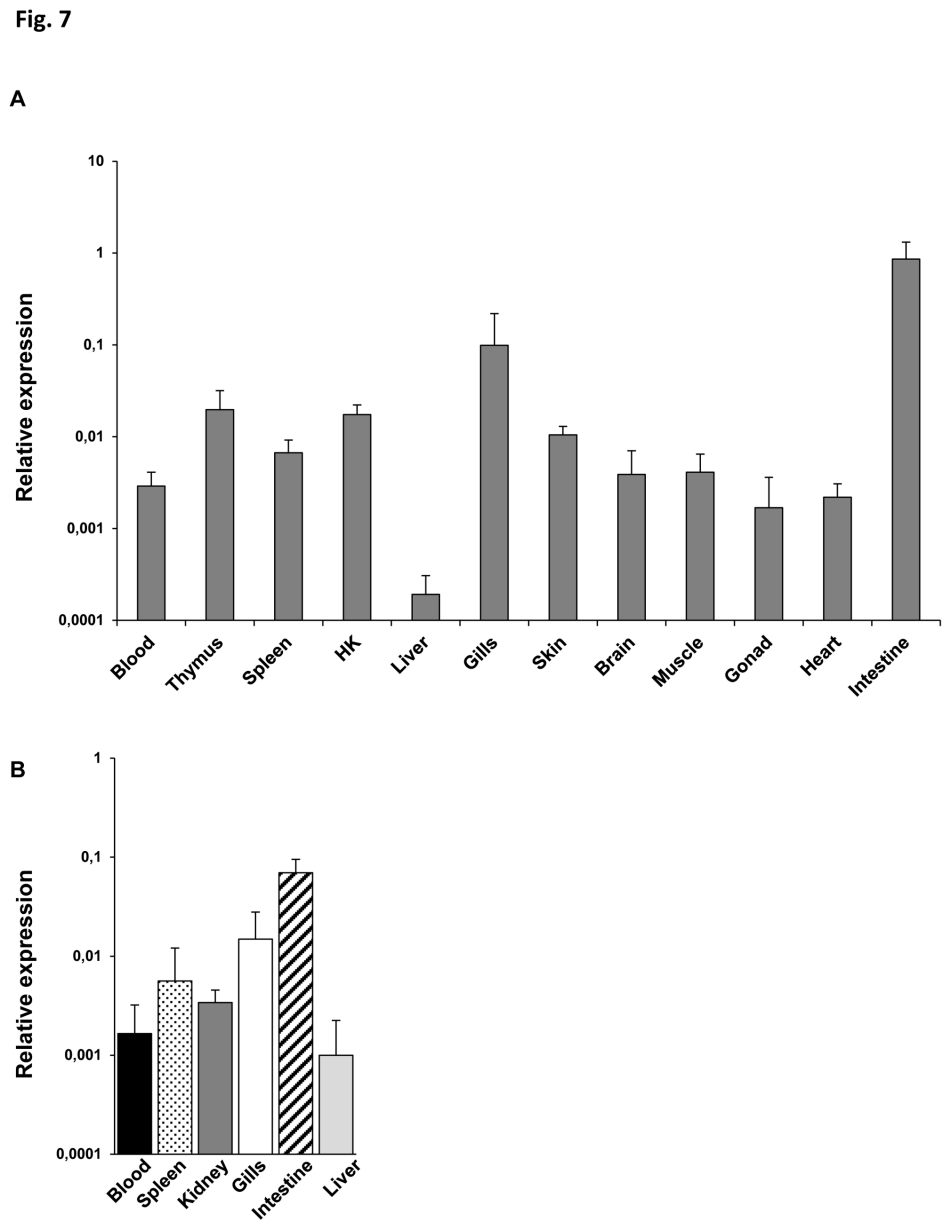
Levels of MS4A transcription in rainbow trout tissues and sorted IgM^+^ cells. A. MS4A mRNA levels measured by real time PCR in different trout tissues. Data are shown as the mean gene expression relative to the expression of an endogenous control (EF-1α) ± SD obtained in 3 naive individuals. B. Levels of transcription of MS4A in sorted IgM^+^ cells from blood, spleen, kidney, gills, intestine and liver. Results are shown as the mean gene expression relative to the expression of EF-1α ± SD for 5 independent experiments.

We first evaluated the relative MS4A mRNA levels in tissue preparations extracted from blood, thymus, spleen, kidney, liver, gills, skin, brain, muscle, gonad, heart and intestine of perfused fish (Figure 7A). The highest concentration of MS4A mRNA was found in intestine followed by gills, thymus and kidney. In contrast, we found very low levels of expression in the liver. Subsequently, we also assessed mRNA levels of MS4A in the different tissue-specific sorted IgM^+^ populations. As shown in Figure 7B, the highest MS4A expression levels were found in IgM^+^ cells from intestine followed by gills and spleen. On the other hand, liver IgM^+^ cells showed the lowest MS4A transcription levels, in correlation to the results obtained in the RNA preparations extracted from total tissues. 

### MS4A, Blimp1, Pax5 and IgM gene expression during trout early stages

**Figure 8:**
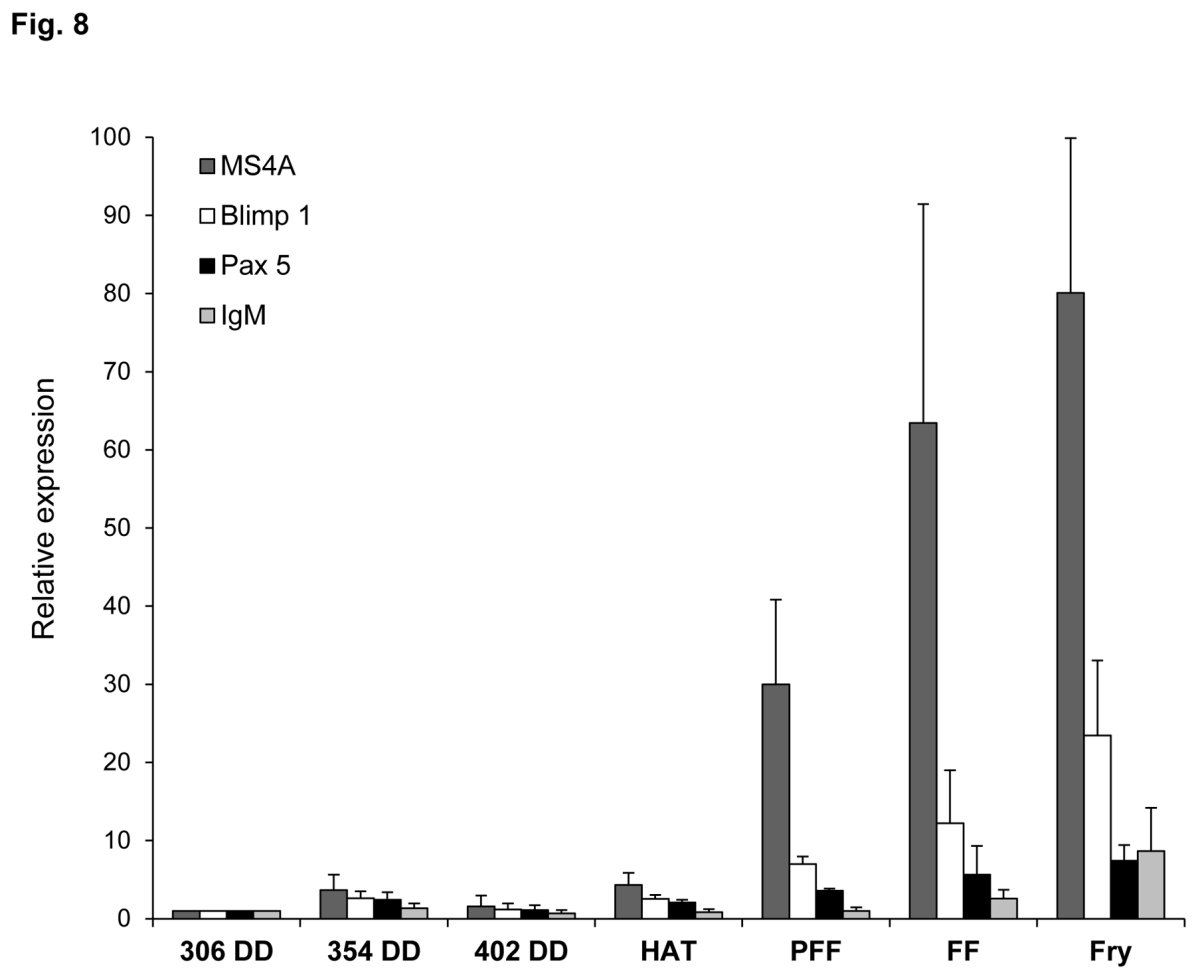
Levels of transcription of B cell markers in early rainbow trout stages. Transcriptional levels of MS4A, Blimp1, Pax5 and IgM during trout early development at different stages. The relative expressions to the endogenous control EF-1α were calculated for each sample and then means values from day 1 were set as 1, and means from the other stages were relative to the first sampling point. Results from 5 individuals per stage ± SD are shown. DD: days degree; HAT: hatching; PFF: pre first feeding ; FF: first feeding.

To describe the transcriptional expression of B cell markers (MS4A, Blimp1, Pax5 and IgM) during trout development we used real time PCR in samples obtained from different early growth stages. As shown in Figure 8, no differences were found in the levels of transcription of the markers analyzed before egg hatching. However, a dramatic increase in MS4A transcription was obtained after hatching, from the first week post-hatching until the third week after the first feeding. Although Blimp1, Pax5 and IgM also suffered an increase during trout development, this was much lower than that observed for MS4A. These results suggest that B cell differentiation takes place during the first weeks after egg hatching and point to a role of trout MS4A in hematopoiesis. 

## Discussion

Although IgM^+^ B cells have been identified in the blood, spleen, kidney, gills and intestine of different fish species since the 1970s, these populations have not been extensively characterized. Thus, in the current work, we have studied the levels of transcription of different immune genes of sorted IgM^+^ cells from all these tissues, characterizing for the first time in trout mRNA levels in sorted cells rather than in complete tissues. We have also included IgM^+^ cells from liver, since, in mammals, analysis of mononuclear cells in this organ revealed that B cells represent as much as half of the intrahepatic lymphocyte population [44]. Our results indicated that IgM^+^ cells in leukocyte preparations from trout liver accounted for 2.7-13.7% of the cells, levels similar to those observed in kidney and higher than those observed in gills and intestine. 

In relation to chemokine receptors, we have observed some important transcriptional differences among IgM^+^ populations. For example, CCR6, CCR7 and CCR13 were only transcribed in sorted IgM^+^ cells from kidney, gills, intestine and liver, but not in IgM^+^ cells from blood or spleen. CCR6 and CCR7 are both expressed in mammalian B cells. In mammals, CCL20, the only ligand for CCR6, is responsible for the recruitment of B cells to intestinal Peyer's patches and isolated lymphoid follicles [45] or to the skin [46]. CCL19 and CCL21 are the ligands for CCR7 in mammals, and are also thought to play a role in B cell trafficking to the intestine. Specifically, CCR7 seems to be responsible for endothelial B-cell adhesion while the actual recruitment to Peyer´s patches appears to be controlled by CXCR5 [47]. Therefore it seems probable that in trout both CCR6 and CCR7 mediate the recruitment of B cells to mucosal tissues while the role of these chemokine receptors in head kidney and liver remain unsolved. This expression pattern is in accordance with our previous results that showed an increased expression of CCR7 mRNAs in IgM^+^ cells from the gut but not from the spleen of trout infected with an intestinal parasite [15]. On the other hand, in this same work, CCR7 mRNA levels were observed in spleen, specifically in T cells, indicating that in spleen T and not B cells transcribe CCR7. In mammals, although CCL20 [48] and CCL21 [49] have been reported in the inflamed liver, most focus has been made on their capacity to recruit T cells, therefore it is difficult to anticipate a role for these receptors differentially expressed in the liver in B cell mobilization. In fact, more studies should be performed to understand the role of liver immune cells in homeostasis and disease. CCR13 is the other chemokine receptor that was not transcribed in spleen nor blood IgM^+^ cells. This molecule, which had been identified on the basis of sequence similarity to CCR3, constitutes a teleost-specific novel lineage of CC chemokine receptors with no equivalent in humans [14]. Consequently, it was renamed CCR13 to avoid wrong ascriptions to mammalian counterparts. Since complete spleen and blood samples do contain important amounts of CCR13 mRNA [14], it seems that other CCR13^+^ cells types different than IgM^+^ cells are present in blood and spleen. The biological role of this teleost-specific receptor remains to be elucidated. Finally, it is noteworthy that intestine and gill IgM^+^ populations have higher transcription levels of all chemokine receptors than those observed in the other tissues, suggesting a higher mobility of these populations. 

It is well established that B cells play a major role in acquired immunity through the production of antigen-specific antibodies, however, their role in innate immunity was not clearly appreciated until TLRs were discovered [24]. In the current work, we found that all the trout sorted IgM^+^ populations transcribed all of the TLRs included in our study, despite the fact that equivalent transcriptional studies in mammals revealed that B cells did not transcribe some of these TLRs. Even though there are important differences in the pattern of TLRs transcribed in different mammalian species, TLR3 and TLR5 were poorly or not identified at all in B cells [25]. Interestingly, trout IgM^+^ cells from all tissues transcribed moderate levels of TLR3 and high levels of TLR5. TLR5 recognizes flagellin, while TLR3 is the receptor for dsRNA and together with TLR4 (not identified in trout), they are the only TLRs that signal through the TRIF pathway and thus induce interferon ß (IFN-ß) [50]. Therefore the transcription of TLR3 in trout IgM^+^ cells reveals a capacity to sense dsRNA and produce IFN, as was described in humans for a specific subset of upper respiratory mucosa B cells [51]. In that study, it was demonstrated that dsRNA triggered not only IFN, but also IgA and IgG through this innate TLR-3 dependent pathway. In the pufferfish fugu (*Takifugu rubripes*), the capacity of TLR3 to sense dsRNA, to recruit TIR-containing adaptor molecule and to consequently induce IFN production has been demonstrated [52], suggesting that a similar mechanism could be present in fish B cells. Although the levels of TLR transcription were very similar among trout IgM^+^ populations, for TLR3, higher mRNA levels were observed in spleen in comparison to other tissues. This was also true for TLR-2 mRNA levels, higher in both spleen and intestine. Agonists of TLR-2, TLR-4 and TLR-9 have a unique capacity to stimulate IL-10 production by naive mouse B cells *in vitro* [53], therefore it might be possible that important subsets of B cells with immunosuppressive capacities are present in both tissues. Additionally, kidney IgM^+^ cells expressed higher levels of TLR-5, TLR-8a2, TLR-9 and TLR-22 than IgM^+^ cells suggesting an important role in antigen sensing and initiation of the immune response. 

Activated B cells and resting B cells express membrane IgM and the transcription factor Pax5. Plasmablasts are down the differentiation pathway and are larger, proliferating, membrane IgM^+^ B cells that produce more secreted IgM. Finally, plasma cells lack both membrane IgM and Pax5, secrete the highest amount of IgM and stop to proliferate [54]. The transcriptional repressor Blimp1 reduces Pax5 levels and shifts Ig expression from the membrane to the secreted form [39]. In this study, we have also observed some important differences in the levels of transcription of Pax5 and Blimp1 and while blood and intestine IgM^+^ cells showed the lowest levels of Pax5 transcription, intestine and gill IgM^+^ cells contained the highest amounts of Blimp1 mRNAs. These results suggest the constitutive presence of IgM-secreting cells in mucosal tissues, as recently reported in humans [55]. In any case, the lack of correspondence between the levels of Blimp1 and Pax5 in some tissues could indicate a further differentiation of subpopulations into various subtypes with differences in their activation state, as already described in the trout kidney [54]. 

Lastly, we report the identification of a member of the MS4A family in trout. Whether this membrane protein is in fact a CD20 ortholog which may be used in the future as a pan-B cell marker for a purification of B cells alternative to purification with anti-IgM antibodies should be further investigated through functional studies. The fact that the MS4A family remains mostly uncharacterized in mammals and that fish MS4A sequences reported are grouped into a single cluster apart from mammalian sequences has lead other authors to suggest that there are no orthologs in fish for mammalian MS4A genes [32]. In the current study, we find levels of transcription among IgM^+^ subpopulations similar to Blimp1 levels in accordance to what might be expected from a CD20 ortholog. However, the ratios of MS4A expression in the sorted IgM^+^ cells in comparison to the negative fraction were always lower than those obtained for Blimp1. In mammals, different members of the MS4A family have been produced by gene duplications and share a sticking conservation of the three transmembrane domains [27], therefore, even though the structural analysis as well as the expression pattern points to the newly described MS4A sequence as an homolog of human CD20, more studies should be performed to determine which MS4A family members are present in teleost fish, and whether their functions are maintained. In any case, the level of expression of this MS4A gene differs in sorted IgM^+^ cells from different origins and is strongly increased through the early developmental stages, results that associate an MS4A family member for the first time in fish to lymphoid cells and hematopoiesis.

In conclusion, we have demonstrated that IgM^+^ cells transcribe a wide range of chemokine receptors and TLRs that suggest an important role in the triggering of innate responses after pathogen sensing. The differences in the patterns and levels of transcription of these genes as well as Pax5 and Blimp1 genes observed among different tissues suggest heterogeneity among IgM^+^ cells. Finally, we have also identified a MS4A gene in trout that is also differentially transcribed in B cells from different tissues as well as throughout development. Although we still don´t know whether these differences correlate to differences in protein expression, the data provided constitute a first step towards the identification of distinct B cell populations in fish.

## Supporting Information

Figure S1 **Flow cytometry dot plots (**A**) and Giemsa staining (**B**) of sorted IgM^+^ cells from the head kidney.** Leukocyte populations obtained after the Percoll gradients were first gated on the basis of their FSC/SSC profile and then on surface IgM expression.
(TIF)

Figure S2 **Alignment of trout TLR2.** Alignment of predicted rainbow trout (OM) TLR2 amino acid sequence deposited in the GenBank (Accession number CCK73195) with potential fully characterized TLR2 ortologs from other fish species. TR: *Takifugu rubripes* (Accession number AAW69370), PO: *Paralichthys olivaceus* (Accession number BAD01046) and EC: *Epinephelus coioides* (Accession number AEB32453).
(TIF)

Figure S3 **Selected rainbow trout CD20-like blast search hits.** The hits were obtained using the predicted protein sequence to search the non-redundant protein database using the DELTA-BLAST Algorithm. All were within the top 16 matches.
(TIF)

Figure S4 **Percentages of identity and divergence among available MS4A sequences.** The sequence obtained for trout MS4A was translated to protein sequences and aligned with available published amino acid sequences for mammals and zebrafish MS4A genes using the ClustalW algorithm. The obtained percent identity (upper right matrix) and divergence (bottom left matrix) value for each sequence pair is shown. Divergence was calculated by comparing sequence pairs in relation to the phylogeny reconstructed by MegAlign, while for the percentages of identity the sequences were compared directly, without accounting for phylogenetic relationships.
(TIF)
