# Supplementary figures and images for: Transcriptional Heterogeneity of IgM+ Cells in Rainbow Trout (Oncorhynchus mykiss) Tissues
Source: PLoS One. 2013 Dec 6;8(12):e82737. doi: 10.1371/journal.pone.0082737 (PMC3855791; doi:10.1371/journal.pone.0082737)

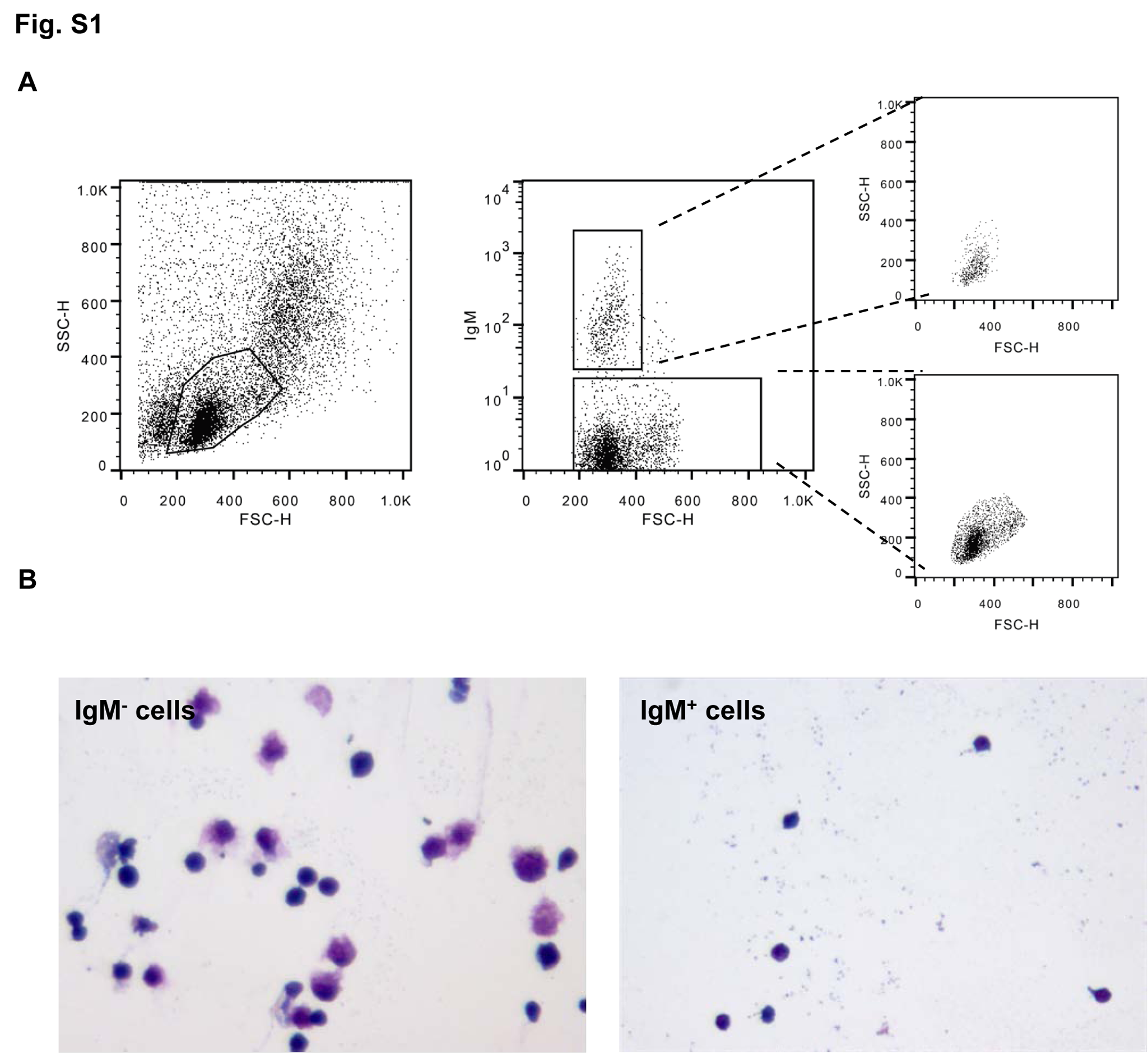

Supplement: Figure S1 — Flow cytometry dot plots (A) and Giemsa staining (B) of sorted IgM+ cells from the head kidney. Leukocyte populations obtained after the Percoll gradients were first gated on the basis of their FSC/SSC profile and then on surface IgM expression. (TIF) [file pone.0082737.s001.tif]

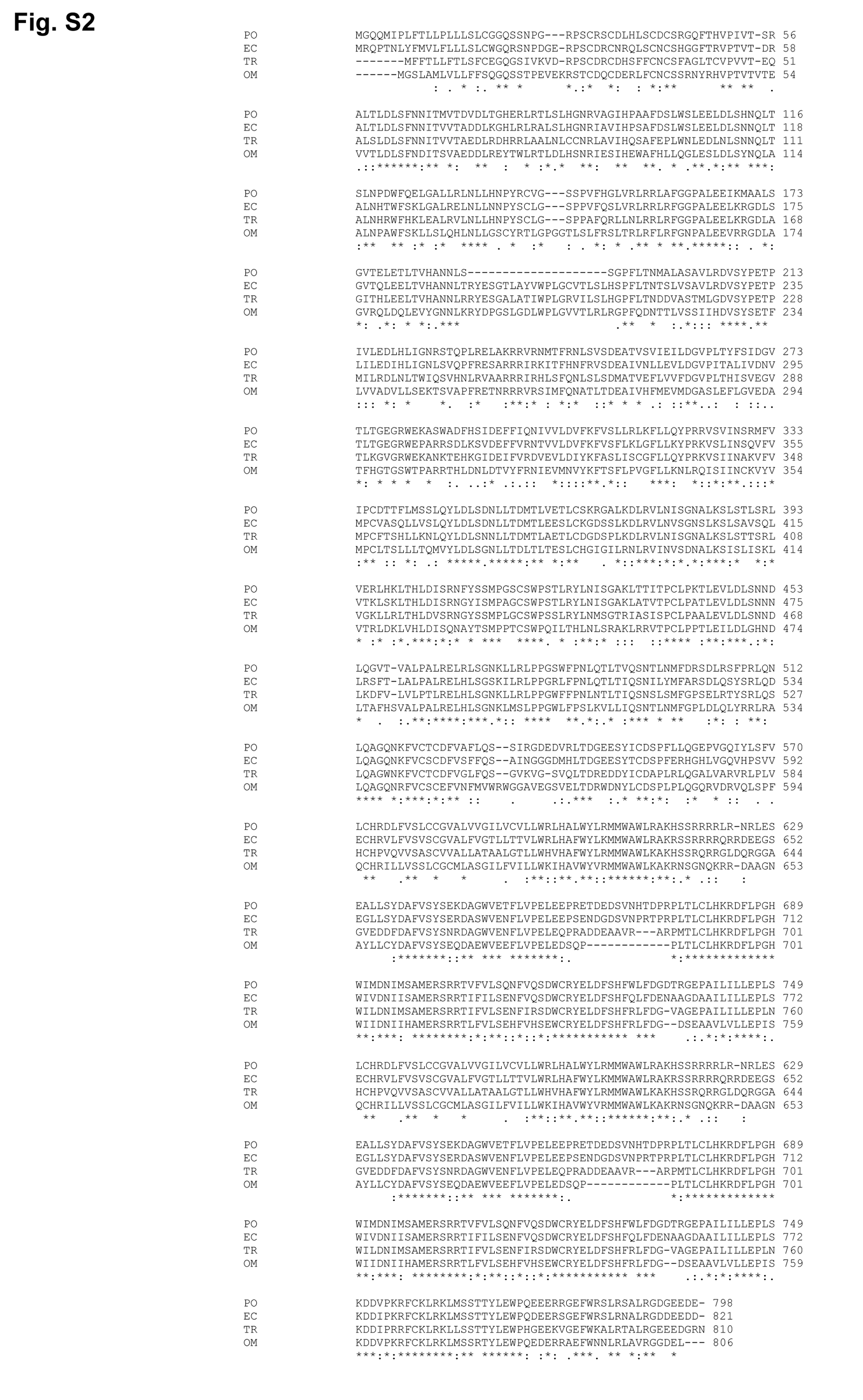

Supplement: Figure S2 — Alignment of trout TLR2. Alignment of predicted rainbow trout (OM) TLR2 amino acid sequence deposited in the GenBank (Accession number CCK73195) with potential fully characterized TLR2 ortologs from other fish species. TR: Takifugu rubripes (Accession number AAW69370), PO: Paralichthys olivaceus (Accession number BAD01046) and EC: Epinephelus coioides (Accession number AEB32453). (TIF) [file pone.0082737.s002.tif]

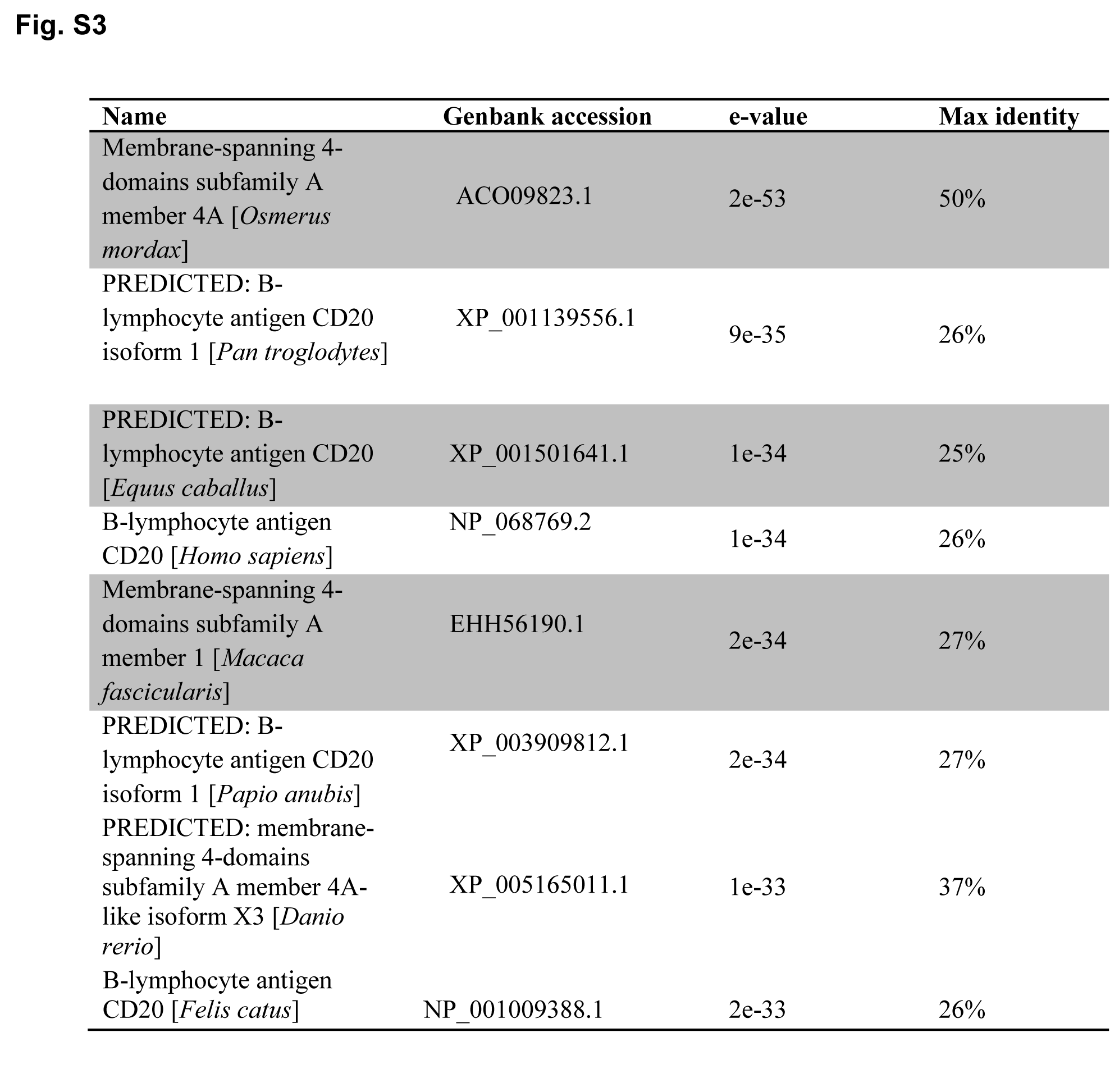

Supplement: Figure S3 — Selected rainbow trout CD20-like blast search hits. The hits were obtained using the predicted protein sequence to search the non-redundant protein database using the DELTA-BLAST Algorithm. All were within the top 16 matches. (TIF) [file pone.0082737.s003.tif]

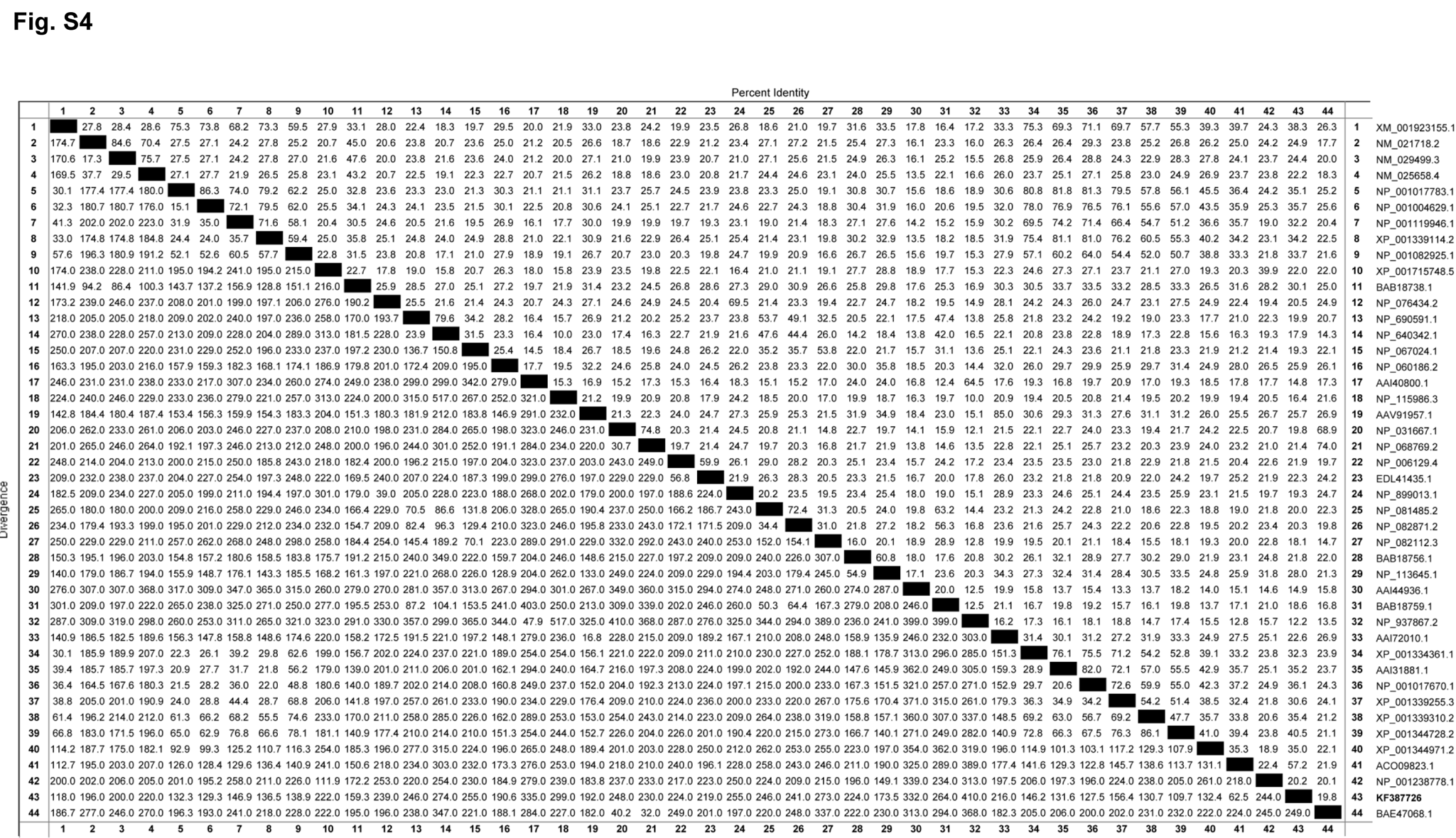

Supplement: Figure S4 — Percentages of identity and divergence among available MS4A sequences. The sequence obtained for trout MS4A was translated to protein sequences and aligned with available published amino acid sequences for mammals and zebrafish MS4A genes using the ClustalW algorithm. The obtained percent identity (upper right matrix) and divergence (bottom left matrix) value for each sequence pair is shown. Divergence was calculated by comparing sequence pairs in relation to the phylogeny reconstructed by MegAlign, while for the percentages of identity the sequences were compared directly, without accounting for phylogenetic relationships. (TIF) [file pone.0082737.s004.tif]
